# Reduced glomerular and elevated tubulointerstitial transglutaminase pathway and its inhibition in a rat model of renal warm ischemia: implications for feline chronic kidney disease

**DOI:** 10.3389/fvets.2025.1520917

**Published:** 2025-07-14

**Authors:** A. C. Sánchez-Lara, M. Maamra, J. L. Haylor

**Affiliations:** ^1^Department of Veterinary Medicine, School of Biological Sciences, Queen's Veterinary School Hospital, The University of Cambridge, Cambridge, United Kingdom; ^2^School of Chemical, Materials and Biological Engineering, University of Sheffield, Sheffield, United Kingdom; ^3^Department of Infection, Immunity and Cardiovascular Disease, Academic Nephrology Unit, Medical School, University of Sheffield, Sheffield, United Kingdom

**Keywords:** glomerular transglutaminase, tubulointerstitial transglutaminase, feline chronic kidney disease, renal warm ischemia, transglutaminase 2 (TG2)

## Abstract

**Introduction:**

Feline CKD is associated with an increase in the pro-fibrotic enzyme, transglutaminase 2 (TG2), in the kidney tubulointerstitium. Hypoxia is pivotal factor for the development of CKD, irrespective of its origin. In cats, tubulointerstitial sclerosis develops without significant glomerular involvement, similar to a rodent model of renal warm ischaemia (RWI).

**Methods:**

Sprague-Dawley rats underwent 60-min renal hilar clamping followed by right nephrectomy with/without intrarenal infusion of a transglutaminase inhibitor (TGI). Renal fibrosis was assessed by immunofluorescence of collagens after 28-days. Extracellular-TG-enzyme activity (eTGact) and extracellular-TG2 protein (eTG2) were measured in both the glomerular and the tubulointerstitial spaces.

**Hypothesis:**

Renal Warm Ischemia (RWI) will induce fibrotic changes and activation of the transglutaminase pathway in both the tubulointerstitial and glomerular compartments, and that treatment with a transglutaminase inhibitor (TGI) will mitigate these effects.

**Results:**

Rats subjected to RWI showed a significant elevation in tubulointerstitial collagen I (1.8-fold), III (4.3-fold), IV (5.5-fold), eTGact (2-fold) and eTG2 (1.9-fold), together with an increase in serum creatinine (2.7-fold). TG inhibition significantly reduced tubulointerstitial collagen I, III, IV, eTGact and eTG2 by 100%, 57%, 90%, 89%, and 91%, respectively, and decreased creatinine levels by 70%. However, RWI in the glomerulus showed a significant reduction in the TG pathway and collagen I and IV.

**Discussion:**

Our findings support a causal link between TG2 and tubulointerstitial fibrosis in rats following RWI. In contrast, the glomerular TG-pathway was suppressed, suggesting a protective mechanism in response to RWI, which may help to explain the lack of glomerular involvement in feline CKD. This rodent model of RWI may be analogous to feline CKD, enabling extrapolation of findings from rodent RWI models to understand renal insult in cats.

## 1 Introduction

Marino et al. (1) showed that half of a randomly selected group of cats had developed some degree of chronic kidney disease (CKD), 30% being over 10 years of age. Despite the high prevalence of feline CKD, drug discovery efforts have remained scarce, highlighting the urgent need to explore novel molecular targets and identify potential diagnostic markers. Animal models in other species with similar functional and histopathological features may facilitate the understanding of the disease.

The major histopathological feature in feline CKD is tubulointerstitial fibrosis, with a low level of glomerulosclerosis (2, 3). Tubulointerstitial fibrosis may be described as the expansion of the extracellular matrix (ECM), mainly due to collagen accumulation. Collagen cross-linking proteins are major factors in both the initiation and the progression of CKD, making interstitial fibrosis resistant to proteolytic degradation leading to progressive collagen deposition that substitutes functional tissue. In human patients with CKD (4) where glomerular scarring is a common histopathological feature, such as in diabetic nephropathy (5), a significant correlation between transglutaminase 2 (TG2) and fibrosis in both the tubulointerstitial and glomerular space has been demonstrated. TG2 has been identified in renal epithelial, endothelial and mesangial cells and secreted when cells are under hypoglycaemic, hypoxic or inflammatory stress (6). Externalized TG2 then interacts with extracellular calcium promoting the formation of an amino bond binding γ-carboxamide glutamic acid and ε-amino group of lysine peptides in collagen fibers. This process accelerates the build-up of extracellular matrix through the creation of a profibrotic and irreversible crosslinking products of epsilon (γ-glutamyl)-lysine dipeptide bonds, which are highly resistant to proteolytic degradation (6).

Sanchez-Lara et al. (7) were the first to describe an association between tubulointerstitial fibrosis and transglutaminase 2 in feline CKD; however, no glomerular scarring or increase in glomerular TG2 was observed. In the domestic cat, an ischaemic or hypoxic insult has been postulated to trigger and promote progression of CKD (8–10).

Hypoxia has been proposed as a common root cause of renal fibrosis, independent of its etiology (11–13). Ischemia reperfusion injury in the cat, induces morphologic changes of acute kidney injury (AKI) which precede the development of CKD, suggesting a hypoxic stimuli can trigger and generate renal fibrosis (9, 10). Recent research has shown that hypoxia-induced mediators of fibrosis such as hypoxia-inducible factor-1 alpha (HIF-1 alpha), matrix metalloproteinases-2 (MMP2),−7 (MMP7), and−9 (MMP9), tissue inhibitor of metalloproteinase-1 (TIMP1), and transforming growth factor-β1 (TGFB1) are upregulated in feline kidneys with CKD supporting the link between hypoxia-fibrosis-CKD in the cat (14). Similar findings in rodent models of RWI have been described (15–17).

The functional and histopathological features to feline CKD may be modeled in the rat following an insult of renal warm ischemia (RWI) (8, 13). We hypothesize that RWI will induce changes in collagen deposition and activation of the transglutaminase pathway in both the tubulointerstitial and glomerular compartments, and that treatment with a transglutaminase inhibitor (TGI) will mitigate these effects.

The aim of the current experiment was to undertake an interventional study using a transglutaminase inhibitor in a rat model of RWI. A major feature of the analytical approach was to assess changes in the TG2 pathway and the development of kidney fibrosis in the glomerular and the tubulointerstitial spaces independently. Rats were subjected to a 60-min period of RWI and kidney tissue analyzed after 28 days following treatment with a transglutaminase inhibitor. TG2 protein and TG enzyme activity were measured in (a) the intra-glomerular mesangium and in (b) the cortical tubulointerstitial space. The development of renal fibrosis was assessed from the deposition of individual collagen proteins.

## 2 Material and methods

### 2.1 Experimental protocol

Male Sprague-Dawley rats (Harlan, UK), 8–10 weeks, with an initial weight of 250–300 g were maintained at 20°C, 45% humidity, light cycle of 12 h and allowed free access to rat food (Harlan Teckland Global, 18% protein rodent diet) and tap water.

For anesthesia induction, rats were placed in an anesthetic chamber with 5% isoflurane for 2–5 min. Following induction, a 0.25 cm strip of ophthalmic ointment was applied to both eyes, and the animal was positioned on a surgical mat. Anesthesia was maintained with 2–3% isoflurane delivered via an anesthetic mask. Anesthetic depth was monitored by visually assessing the respiratory rate. Analgesia was provided through an intramuscular injection of buprenorphine (50 μg/kg) into the left hind limb. To ensure thermal stability during anesthetic procedures, particularly for the warm ischaemic stimulus, body temperature was maintained between 36 and 37°C using a thermostatically controlled heating blanket, with continuous monitoring via a rectal probe.

Experimental procedures were approved by the Animal Welfare and Ethical review body (University of Sheffield) and carried out in accordance with the Animals Scientific Procedures Act (United Kingdom 1986).

Rats were divided into three experimental groups. Control rats (Nx, *n* = 5) were sham operated and subjected to right nephrectomy at day 7. The two ischaemic groups, RWI (*n* = 5) and RWI+TGI (n = 6), consisted of rats subjected to intrarenal cannulation and placement of a subcutaneous osmotic minipump (day-3). The subcutaneous pumps were loaded with 2 ml of either NaCl 0.9% (vehicle) or vehicle + transglutaminase inhibitor (50 mmol/L, TGI, 1,3-dimethyl-2[(2-oxopropyl)thio]imidazolium, DOO3, Zedira, Germany), delivered at 100 μg/kg/h for 31 days. Osmotic minipumps were filled under aseptic conditions using a laminar flow hood. The pumps were manipulated using sterile gloves over a sterile surface.

The experimental sample size was established based on previous pilot studies conducted at the Academic Nephrology Unit at the University of Sheffield. No power calculation was performed for this study; however, the chosen sample size was deemed sufficient to provide meaningful biological insights while adhering to the principles of the 3Rs (Replacement, Reduction, and Refinement) in animal research.

All three groups underwent three anesthetic episodes:

The first episode occurred on **day-3**, during which the disease groups (RWI and RWI+TGI) underwent intrarenal cannulation and placement of a subcutaneous implant. In contrast, the sham-operated control group (Nx) underwent only left flank skin and muscle incisions, with an anesthetic duration matched to that of the disease groups (30 min).

The second episode took place on **day 0**, where rats in the disease groups (RWI and RWI+TGI) were subjected to 60 min of left renal hilar clamping. The Nx group underwent anesthesia for the same duration without undergoing hilar clamping.

**Figure 1:**
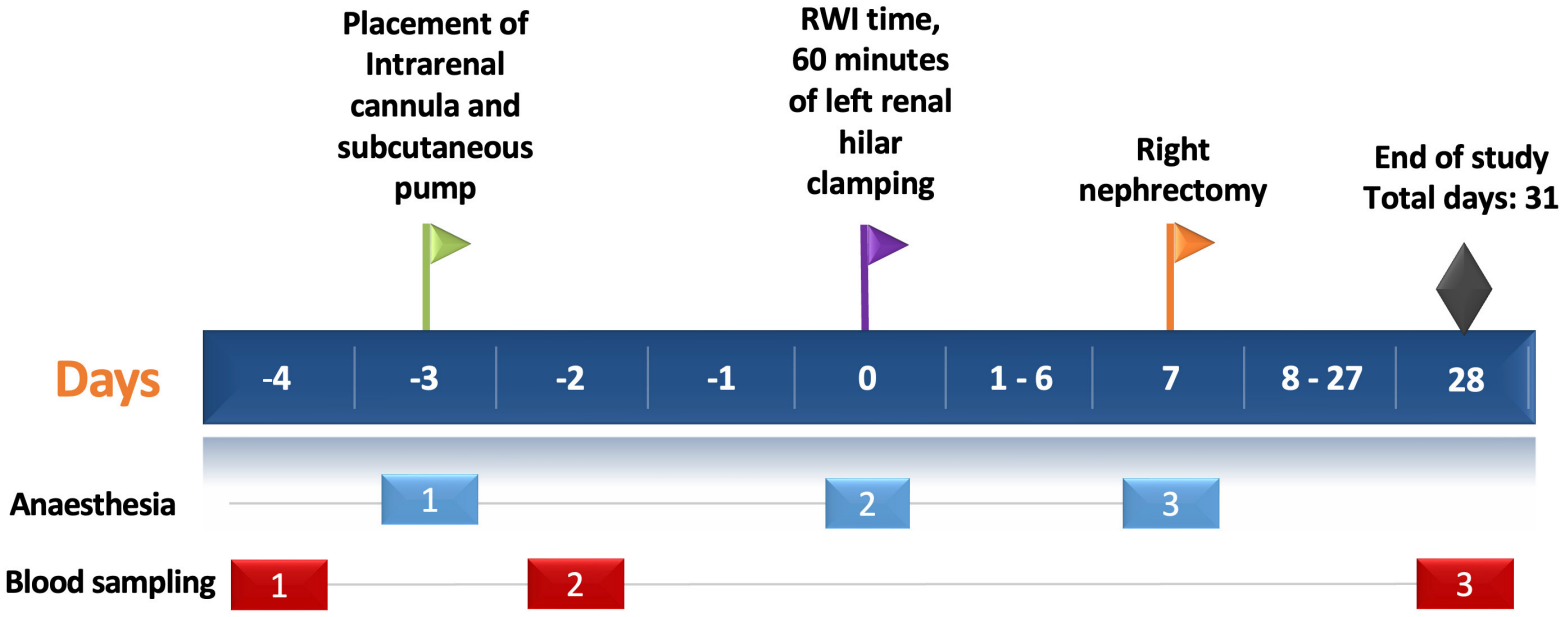
Experimental protocol timeline. The horizontal blue bar depicts the days of the experimental protocol (31 days in total). The green flag shows the day of intrarenal cannulation in the left kidney (day-3). The purple flag shows the day of left renal hilar clamping for 60 min—RWI time (day 0). The yellow flag shows the time of the right nephrectomy (day 7). Black diamond shows the end of the study. Light blue and red rectangles depict the episodes of anesthesia (three episodes) and blood sampling (three episodes), respectively. RWI, Renal Warm Ischaemia.

The third episode occurred on **day 7**, during which all three groups underwent right nephrectomy, Figure 1.

The animal experimentation period lasted 31 days, from the insertion of the intrarenal cannula (day-3) to the study's conclusion on day 28. Following euthanasia, kidney tissue was immediately collected. Two quarters of the tissue were placed in separate containers with 10% formalin, while the remaining two quarters were stored in different containers in a biorepository bank at −80°C.

Technical failure of the osmotic pump system at the end of the experimental time was set as exclusion criterion. One rat and two rats from groups RWI and RWI+TGI originally numbered (RWI, *n* = 6) and (RWI+TGI, *n* = 8), respectively, were removed from the study due to this reason. No inclusion criteria were set for this experimental study.

To minimize potential confounders such as treatment order, measurement bias, and environmental influences, several strategies were implemented in this study. Upon arrival, rats were randomly selected from their cages and assigned a unique identification number by marking the base of their tail with a permanent marker, ensuring unbiased allocation. Markings were reapplied as needed to maintain clear identification throughout the study. Surgical interventions were consistently performed in the early morning to standardize conditions and reduce variability related to circadian influences. Additionally, cages were rotated within the racks at regular intervals to mitigate potential environmental confounders, such as variations in lighting, temperature, or airflow distribution. These measures were taken to ensure that no systematic bias affected the outcomes of the study.

There was no randomization to allocate experimental groups using computer-based software. The allocation of rats per group was based on choosing rats from randomly selected cages. The first author was aware of the group allocation during animal experimentation; however, the analytical samples were blinded to the person doing the experimental analysis.

### 2.2 Intrarenal infusion

A sterile polyethylene cannula (12 cm length, 0.58 mm diameter) was attached to a sterile osmotic minipump (Alzet, 2ML4, US), the end was heat sealed, and 12 fenestrations were made 5 mm before the sealed end, with a 1.5 cm total fenestrated area. Three days before RWI, the fenestrated cannula passed through the kidney longitudinally from caudal to cranial pole with the aid of an intravenous catheter to achieve a straight and quick renal insertion. The cannula was fixed to the kidney by glued silk ligatures placed on the cranial and caudal poles of the renal parenchyma. The intrarenal infusion of transglutaminase inhibitors was performed as previously described by Huang et al. (18). This technique has been proved to distribute the TG inhibitor evenly in both longitudinal and transverse planes of the kidney (18). The minipump was placed in a subcutaneous dorsal pocket as previously described (19) allowing accurate intrarenal vehicle/drug administration (20).

### 2.3 Renal warm ischemia

The left kidney was exposed via a flank incision. The renal artery and vein were clamped for 60 min (day 0) with a 45° angled vascular bulldog clamp, (Vascu-statt II, SCALAN International). Visual evaluation of kidney surface color from reddish (oxygenated kidney) to dark brown (hypoxemic kidney) was used to indicate an effective clamping of renal hilum. Total recovery of kidney color was obtained within 2 min of clamp removal. At day 7, right nephrectomy was performed through a right flank incision.

The rationale for performing right nephrectomy on day 7 was primarily to enhance rat survival while maintaining adequate renal function during the acute injury phase affecting the left kidney. A pilot study establishing the current 60 min renal warm ischemia (RWI) model demonstrated a 45% mortality rate when right nephrectomy was performed concurrently with RWI. In contrast, a separate study reported a reduced mortality rate of 20–25% when nephrectomy was delayed until day 5 (21). An alternative approach, involving bilateral renal warm ischemia without nephrectomy, resulted in a lower incidence of glomerular and tubulointerstitial injury (13). Several studies have investigated RWI in rats, varying in ischemia duration (45–60 min), the number of affected kidneys (one or both), and the timing of contralateral nephrectomy (same day, day 5, or day 10). Each study demonstrated different degrees of renal injury with histopathological reproducibility (13, 21–25). Notably, a rodent model developed by the last author (JLH) identified normothermia, rather than hypothermia, during RWI as a critical factor influencing the severity of histological renal damage in a similar rodent model (26). Considering survival rates, previous studies employing similar models, and prior longitudinal histopathological assessments evaluated at various time points over 4 months using hematoxylin and eosin (H&E) and Masson's trichrome staining—the 60 min RWI model with contralateral nephrectomy on day 7 was selected.

All surgical interventions, including placement of osmotic pump and intrarenal canula, were conducted by clipping the rat's abdominal flank (left or right, depending on the intervention) delimiting a 3 × 3 cm surgical area, followed by aseptic preparation using three successive applications of 2% chlorhexidine. Additionally, the palmar and plantar surfaces of the limbs were meticulously cleaned with 2% chlorhexidine to minimize bacterial contamination. All procedures were performed by ASL using surgical scrubs and masks. Sterile gloves were used prior surgical hand scrubbing protocol with chlorhexidine. The surgeries were carried out in a designated laboratory animal surgical suite using a surgical microscope equipped with a camera (Olympus).

Sterile, autoclaved surgical instruments and materials per rat were used throughout, including sterile gloves, swabs, cotton buds, and fenestrated blue sterile drapes to ensure appropriate coverage and asepsis of the animal during the procedure.

### 2.4 Renal function

Serum creatinine was measured by Jaffe method (Synchron System, Beckman Coulter Inc., US). Rat urine albumin was measured by enzyme-linked immunosorbent assay (E111-125, Bethyl Laboratories, US). Three blood samples were collected from each rat at specific time points. The first sample was obtained 24 h before intrarenal cannulation (day-4), while the second was collected 24 h after the procedure. The final blood sample was collected at the end of the study on day 28, Figure 1.

### 2.5 Tissue analysis

#### 2.5.1 Extracellular transglutaminase enzyme activity

Eight micrometer thick kidney cryostat sections were blocked in 50 mM Tris buffer (pH 7.4) supplemented with 5% goat serum, 10 mM EDTA, streptavidin 5 μg/mL with protease inhibitors (*Complete* Protease Inhibitor Cocktail *Tablets*, Roche, US) in Triton 0.01% for 10 mins. Slides were then washed and incubated (1 h, 37°C) in 50 mM Tris buffer with 0.5 mM biotin cadaverine (Molecular Probes, Invitrogen, US), 5 mM dithiothreitol (DTT) and 5 mM CaCl_2_ (27). Controls were incubated with 10 mM EDTA, 100 μM TG inhibitor using 1, 3-dimethyl-2[(oxopropyl) thio]-imidazolium chloride (D003, Zedira, Germany) or anti-TG2 mouse monoclonal (CUB7402, Abcam, UK). Sections were washed, fixed with cold acetone, air dried and blocked with 3% BSA (4°C, overnight). Sections were then probed with streptavidin Alexa red fluor conjugate 594 (1:300, Invitrogen, US) in 3% BSA (2h, 37°C), washed and mounted using MOWIOL-DAPI (DAPI) mounting media. Twelve cortical fields 400 × (IGMA) and 200 × (tubulointerstitial) were captured and subjected to multiphase image analysis (7). The fluorescent TG activity index was determined calculating the intense Alexa red (TG activity)/DAPI (nuclei) ratio of the total field (18).

#### 2.5.2 Extracellular transglutaminase 2 protein

Eight micrometer thick kidney cryostat sections were incubated with 5% bovine serum albumin in 0.01% Triton X-100 (room temperature, 10 min), followed by overnight (4°C) incubation with a rabbit polyclonal TG2 antibody (1:100, Abcam, Ab421, US). Slides were washed and fixed in cold acetone. TG2 was revealed using a secondary goat anti-rabbit IgG labeled with Alexa red (1:400, Invitrogen, A. Fluor 594, US) (18). For negative controls, only secondary antibody was used. Sections were washed and mounted using MOWIOL-DAPI (DAPI) mounting media. Twelve cortical fields 400 × (IGMA) and 200 × (tubulointerstitial) were captured and subjected to multiphase image analysis (7). TG2 quantification was determined calculating the intense Alexa red (TG2)/DAPI (nuclei) ratio of the total field (18).

#### 2.5.3 Kidney collagens

Eight micrometer thick kidney cryostat sections were incubated with 5% bovine serum albumin in 0.01% Triton X-100 (room temperature, 10 min), followed by 4°C overnight incubation with a rabbit monoclonal anti-collagen I antibody (1:100, Abcam, Ab 34717, US), a goat polyclonal anti-collagen III antibody (1:10, Southern Biotech, 1330-01, US) or a rabbit polyclonal anti-collagen IV (1:35, MP Biomedicals 08681241). Slides were then washed and fixed in cold acetone. Collagens I and IV were revealed using a secondary antibody labeled with FITC swine polyclonal anti-rabbit (1:15, Dako, F0054, US). Collagen III was revealed using a secondary antibody labeled with FITC; a rabbit anti-goat IgG (1:150, Vector, FI5000, US) (18). For negative controls, only secondary antibodies were used. Sections were washed and mounted using MOWIOL-DAPI (DAPI) mounting media. Twelve cortical fields 400 × (IGMA) and 200 × (tubulointerstitial) were captured using an F-View II camera (Soft Imaging Systems, Muenster, Germany) and subjected to multiphase image analysis (7). Collagen quantification was determined calculating the intense FITC (collagens)/DAPI (nuclei) ratio of the total field (18).

#### 2.5.4 HIF-2 alpha

Five micrometer paraffin embedded sections of three tissue samples from the RWI group were deparaffinized and rehydrated using standard protocols. Sections were blocked with 5% (w/v) BSA in PBS for 1 h at room temperature by followed by 4°C overnight incubation with a rabbit polyclonal anti-HIF-2 alpha/EPAS1 antibody (1:100, Novus Biologicals, NB100-122, US). HIF-2 alpha was revealed using a secondary antibody labeled with Alexa fluor plus 488; a goat anti-rabbit IgG (Invitrogen, A10034). For negative controls, only secondary antibody was used. Sections from three RWI kidneys were then washed and mounted using MOWIOL-DAPI (DAPI) mounting media. A cortical and medullar fields 200 × were captured using an F-View II camera (Soft Imaging Systems, Muenster, Germany).

### 2.6 Statistical analysis and assumptions

**Figure 2:**
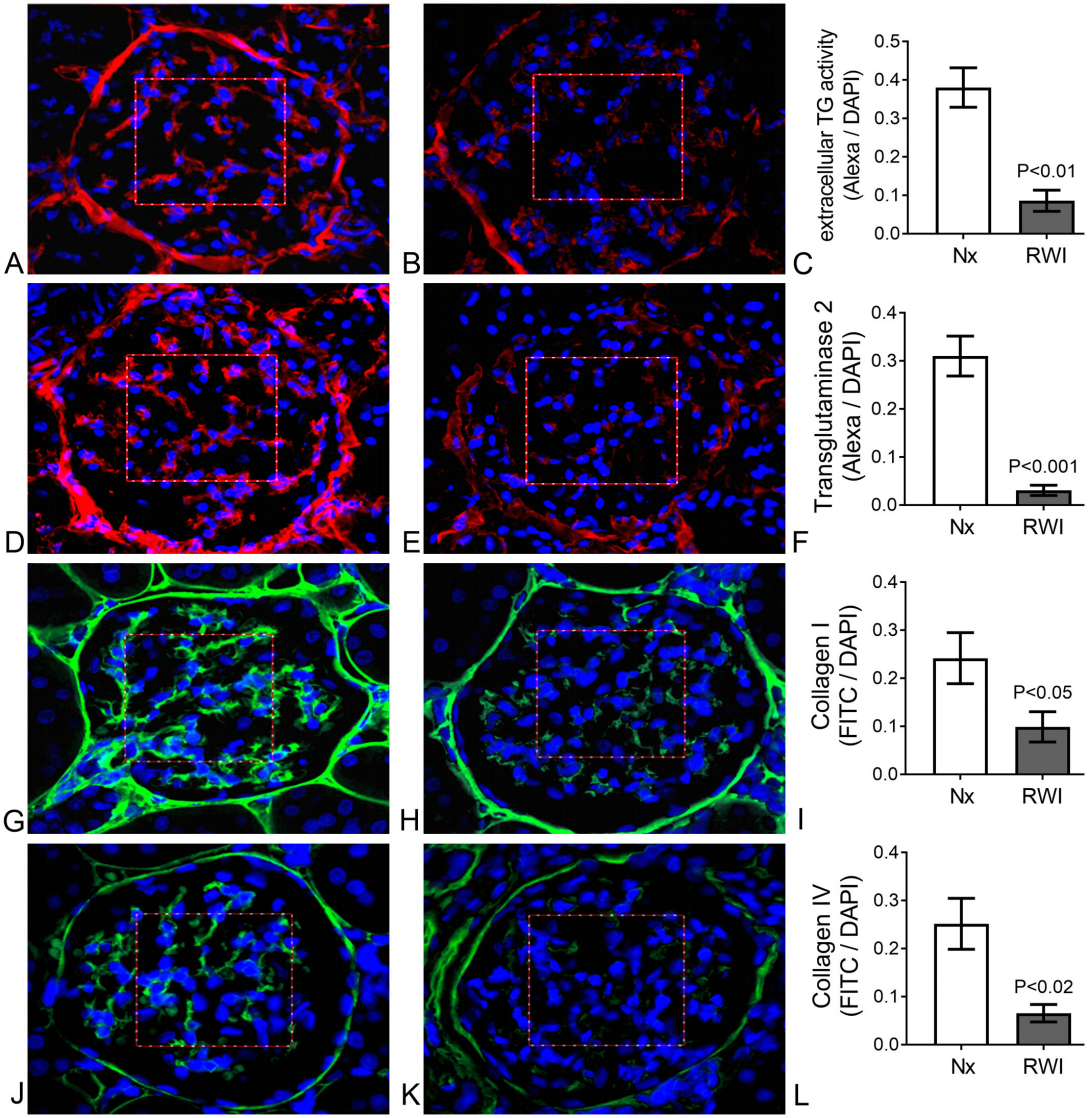
**(A)** Sham operated representative glomerulus. Evident extracellular fluorescence (red) in the IGMA delimited by a squared red frame. Cell nuclei are shown in blue. TG enzyme activity. **(B)** RWI subjected representative glomerulus. Mild extracellular fluorescence (red) in the IGMA delimited by a squared red frame. Cell nuclei are shown in blue. TG enzyme activity. **(C)** Significantly lower Alexa/DAPI ratio in IGMA from RWI rats (*n* = 5) compared to sham operated rats (*n* = 5), determined by extracellular TG activity. Vertical lines on columns are ± SEM. **(D)** Sham operated representative glomerulus. Evident extracellular fluorescence (red) in the IGMA delimited by a squared red frame. Cell nuclei are shown in blue. TG2 immunofluorescence. **(E)** RWI subjected representative glomerulus. Mild extracellular fluorescence (red) in the IGMA delimited by a squared red frame. Cell nuclei are shown in blue. TG2 immunofluorescence. **(F)** Significantly lower Alexa/DAPI ratio in IGMA from RWI rats (*n* = 5) compared to sham operated rats (*n* = 5), determined by extracellular TG2 protein. Vertical lines on columns are ± SEM. **(G)** Sham operated representative glomerulus. Evident extracellular fluorescence (green) in the IGMA delimited by a squared red frame. Cell nuclei are shown in blue. Collagen I immunofluorescence. **(H)** RWI subjected representative glomerulus. Mild extracellular fluorescence (green) in the IGMA delimited by a squared red frame. Cell nuclei are shown in blue. Collagen I immunofluorescence. **(I)** Significantly lower FITC/DAPI ratio in IGMA from RWI rats (*n* = 5) compared to sham operated rats (*n* = 5), determined by collagen I by immunofluorescence. Vertical lines on columns are ± SEM. **(J)** Sham operated representative glomerulus. Evident extracellular fluorescence (green) in the IGMA delimited by a squared red frame. Cell nuclei are shown in blue. Collagen IV immunofluorescence. **(K)** RWI subjected representative glomerulus. Mild extracellular fluorescence (green) in the IGMA delimited by a squared red frame. Cell nuclei are shown in blue. Collagen IV immunofluorescence. **(L)** Significantly lower FITC/DAPI ratio in IGMA from RWI rats (*n* = 5) compared to compared to sham operated rats (*n* = 5), determined by collagen IV by immunofluorescence. Vertical lines on columns are ± SEM.

Data analysis and presentation were performed using Prism 5 software (GraphPad, La Jolla, CA). Data are shown as mean ± SEM. The cross-sectional tubulointerstitial and glomerular measurements were analyzed by one-way ANOVA followed by Bonferroni's multiple comparisons test to compare RWI, RWI+TGI, and Nx groups. For glomerular analysis between Nx and RWI groups depicted in Figures 2C, F, I, L, cross-sectional measurements were analyzed by unpaired two-tailed Student's *t*-test. To evaluate group correlations, *r*^2^ was calculated by linear regression analysis. Variables included in correlation analysis were preselected based on their biological relevance. The statistically significant level was defined as *P* < 0.05.

Assumptions for parametric tests across the three groups were assessed using the Shapiro-Wilk test for normality. Violations of normality were identified in mesangial collagen III and Masson's trichrome staining (MTS). Consequently, mesangial collagen III and MTS data were analyzed using the non-parametric Kruskal-Wallis test, confirming that these variables were not normally distributed.

When comparing mesangial parameters between the Nx and RWI groups, normality was verified for MTS, collagen I, collagen IV, TG2, and TG activity, except for collagen III. Due to its non-normal distribution, mesangial collagen III was analyzed using Welch's *t*-test, which did not yield statistical significance, further supporting the lack of normality.

Levene's test for homogeneity of variances was performed using Microsoft Excel by calculating a one-way ANOVA on the absolute deviations from the group means. Homogeneity for all parameters was confirmed except for mesangial and tubulointerstitial MTS. As a result, mesangial and tubulointerstitial MTS data were excluded from the study, as their variability prevented the attribution of differences to the interventions (renal warm ischemia and transglutaminase inhibitor).

For regression analysis, residuals were tested for normality via Q–Q plots and Shapiro–Wilk test, while homoscedasticity was satisfied by visual examination of the residual plot.

## 3 Results

### 3.1 Kidney function

**Figure 3:**
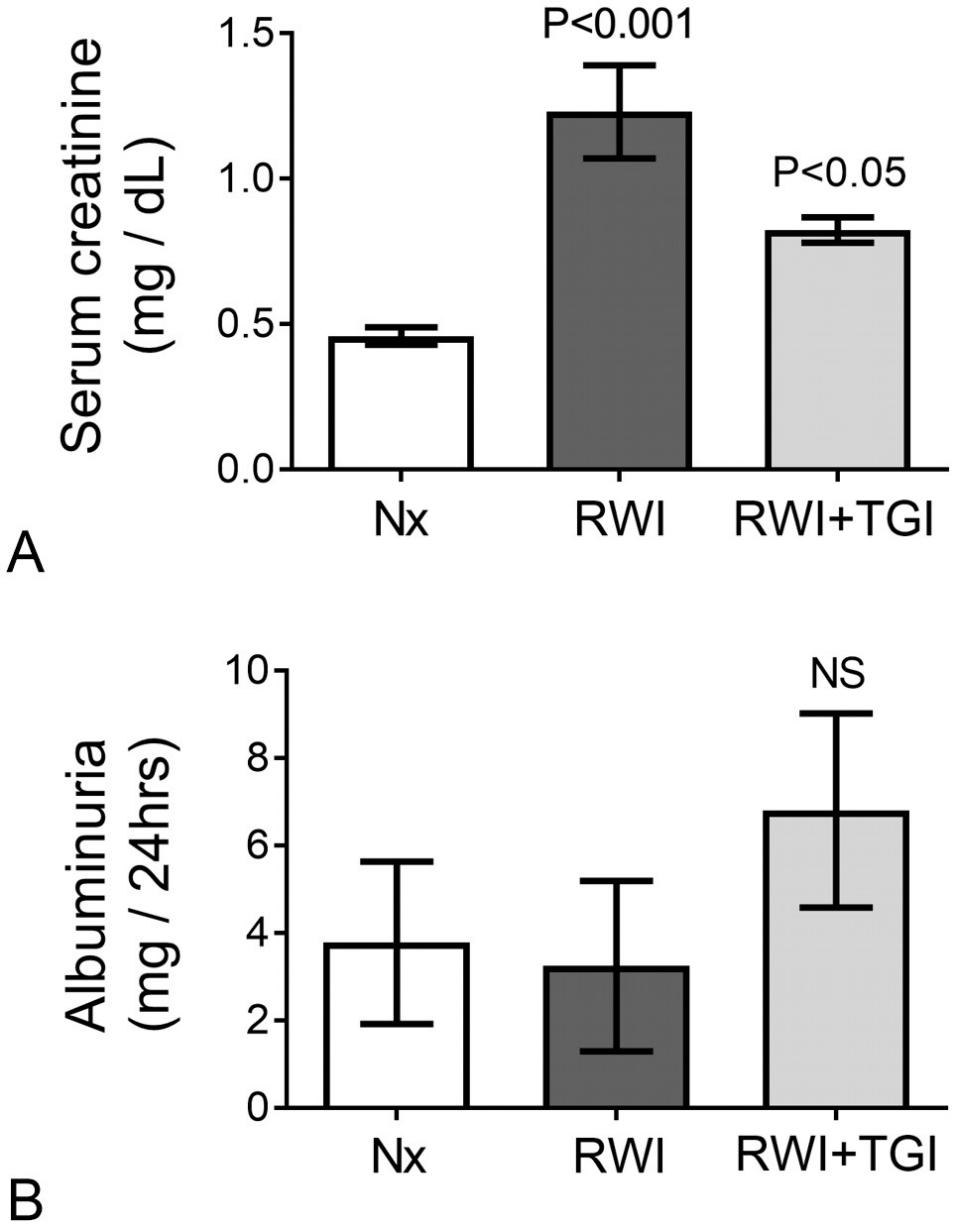
**(A)** Following 28 days of disease progression, the RWI group showed a significant elevation of serum creatinine with significant reduction when treatment was employed (Nx, *n* = 5; RWI, *n* = 5 and RWI+TGI, *n* = 6). Vertical lines on columns are ± SEM. **(B)** No significant changes in albumin excretion were observed in experimental groups (Nx, *n* = 5; RWI, *n* = 5 and RWI+TGI, *n* = 6) after 28 days of RWI. Vertical lines on columns are ± SEM. *P* < 0.05, RWI+TGI compared with RWI at day 28. *P* < 0.001, RWI group compared with Nx group.

Prior to RWI, no difference in serum creatinine was detected either before, day-4 (Nx, 0.32 ± 0.04; RWI, 0.33 ± 0.1; RWI+TGI, 0.32 ± 0.01 mg/dL) or 24 h after intra-renal cannulation, day-2 (Nx, 0.34 ± 0.02; RWI, 0.38 ± 0.04; RWI+TGI, 0.4 ± 0.07 mg/d). Twenty-eight days after the RWI, serum creatinine was significantly elevated with modest but still significant recovery in renal function with the TG inhibitor (Nx, 0.30 ± 0.04; RWI, 1.23 ± 0.16; RWI+TGI, 0.82 ± 0.04 mg/dL, RWI compared with Nx group, *P* < 0.001; and RWI+TGI compared with RWI at day 28, *P* < 0.05, Figure 3A). Nx, *n* = 5; RWI, *n* = 5; RWI + TGI, *n* = 6.

Albumin excretion did not differ between the rat groups when measured on either day 0 (Nx, 0.9 ± 0.7; RWI, 0.47 ± 0.18; RWI+TGI, 0.13 ± 0.1 mg/24 h) or day 28 (Nx, 3.7 ± 1.8; RWI, 3.2 ± 1.9; RWI+TGI, 6.8 ± 2.2 mg/24 h, Figure 3B).

### 3.2 Intraglomerular mesangial TG pathway

Representative examples of glomerular immunofluorescence (Alexa red) for Nx and RWI groups and a histogram comparing both groups are shown for TG enzyme activity in Figures 2A—C, respectively; and TG2 protein in Figures 2D–F, respectively. Cell nuclei, detected by DAPI, are shown in blue. RWI was associated with a lower level of glomerular transglutaminase *in situ* activity (Nx, 0.38 ± 0.05; RWI, 0.09 ± 0.027 Alexa/DAPI ratio, *P* < 0.0001), which remained unaffected by the TGI (RWI+TGI, 0.08 ± 0.03 Alexa/DAPI ratio). RWI was also associated with a significantly lower level of glomerular TG2 protein of 85% compared to Nx (Nx, 0.31 ± 0.04; RWI, 0.03 ± 0.01 Alexa/DAPI ratio, *P* < 0.001), which remained unaffected by TGI (RWI+TGI, 0.05 ± 0.02 Alexa/DAPI ratio). Nx, *n* = 5; RWI, *n* = 5; RWI + TGI, *n* = 6.

### 3.3 Intraglomerular mesangial collagens

Representative examples of glomerular collagen immunofluorescence (Green-FITC) for Nx and RWI groups and a histogram comparing both groups are shown for collagen I in Figures 2G–I, respectively; and collagen IV in Figures 2J–L, respectively. Cell nuclei, detected by DAPI, are shown in blue. Following RWI, the intraglomerular mesangial collagen I was reduced by some 60% (Nx, 0.23 ± 0.03; RWI, 0.1 ± 0.06 FITC/DAPI ratio, *P* < 0.05), which remained unaffected by the TGI (RWI+TGI, 0.07 ± 0.03 FITC/DAPI ratio). Intraglomerular mesangial collagen IV was reduced by some 80% in the RWI group compared to the Nx group (Nx, 0.25 ± 0.05; RWI, 0.06 ± 0.02 FITC/DAPI ratio, *P* < 0.01), which remained unaffected by TGI (RWI+TGI, 0.04 ± 0.004 FITC/DAPI ratio). Intraglomerular collagen III however remained unchanged for the three groups (Nx, 0.004 ± 0.0001; RWI, 0.002 ± 0.001; RWI+TGI, 0.016 ± 0.007). Nx, *n* = 5; RWI, *n* = 5; RWI + TGI, *n* = 6.

### 3.4 Tubulointerstitial transglutaminase pathway

#### 3.4.1 Tubulointerstitial transglutaminase activity *in situ*

**Figure 4:**
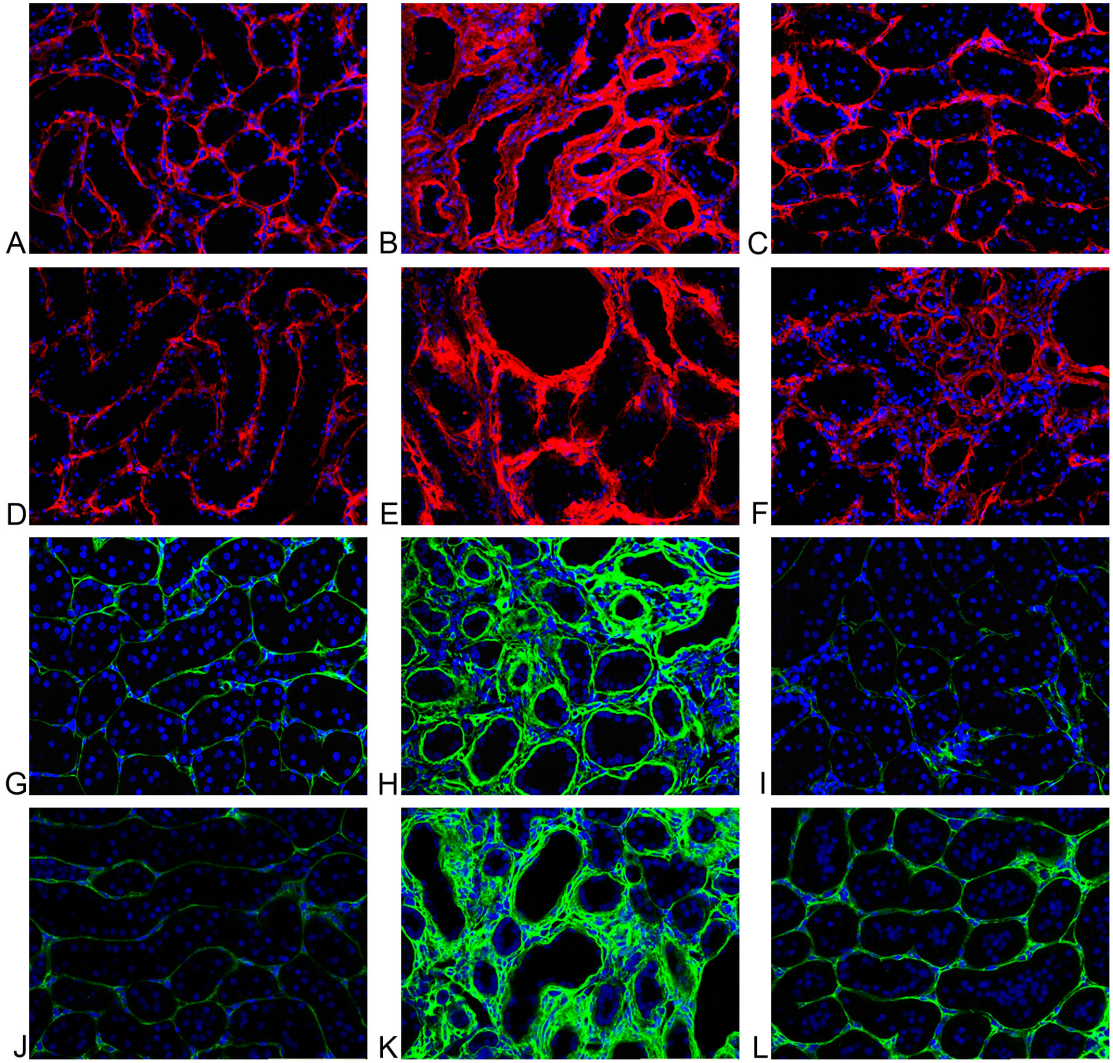
**(A)** Sham operated representative tubulointerstitial area. Minimal extracellular fluorescence (red); cell nuclei in blue. TG activity. **(B)** RWI representative tubulointerstitial area. Increase of extracellular activity (red) and tubular dilation; cell nuclei in blue. TG activity. **(C)** RWI+TGI representative tubulointerstitial area. Mild extracellular fluorescence (red); cell nuclei in blue. TG activity. **(D)** Sham operated representative tubulointerstitial area. Minimal extracellular fluorescence (red); cell nuclei in blue. TG2. **(E)** RWI representative tubulointerstitial area. Increase extracellular activity (red) and tubular dilation; cell nuclei in blue. TG2. **(F)** RWI+TGI representative tubulointerstitial area. Mild extracellular fluorescence (red); cell nuclei in blue. TG2. **(G)** Sham operated rat representative tubulointerstitial area. Minimal extracellular fluorescence (green); cell nuclei are shown in blue. Immunofluorescent staining for collagen I. **(H)** RWI representative tubulointerstitial area. Increased extracellular immunofluorescence (red); cell nuclei are shown in blue. Immunofluorescent staining for collagen I. **(I)** RWI+TGI representative tubulointerstitial area. Minimal extracellular fluorescence (green); cell nuclei are shown in blue. Immunofluorescent staining for collagen I. (J) Sham operated rat representative tubulointerstitial area. Minimal extracellular fluorescence (green); cell nuclei are shown in blue. Immunofluorescent staining for collagen III. **(K)** RWI representative tubulointerstitial area. Increased extracellular immunofluorescence (green); cell nuclei are shown in blue. Immunofluorescent staining for collagen III. **(L)** RWI+TGI representative tubulointerstitial area. Minimal extracellular fluorescence (green); cell nuclei are shown in blue. Immunofluorescent staining for collagen III.

Representative examples of the tubulointerstitial TG activity for Nx, RWI, and RWI+TGI groups are shown in Figures 4A–C, respectively. Cell nuclei, detected by DAPI, are shown in blue. RWI was associated with a two-fold increase in TG activity (Nx, 0.10 ± 0.017; RWI, 0.19 ± 0.02 Alexa/DAPI ratio, *P* < 0.01). TGI significantly reduced the increase in TG activity by 89% to values which showed no statistical difference from the Nx control group (RWI+TGI, 0.12 ± 0.013 Alexa/DAPI ratio, *P* < 0.05). Nx, *n* = 5; RWI, *n* = 5; RWI + TGI, *n* = 6.

Tubulointerstitial TG enzyme activity gave significant but low-moderate linear correlations with tubulointerstitial fibrosis determined by individual collagens, collagen I (*r*^2^ = 0.29, *P* < 0.05), collagen III (*r*^2^= 0.34, *P* < 0.02) and collagen IV (*r*^2^= 0.41, *P* < 0.01).

#### 3.4.2 Tubulointerstitial transglutaminase 2 protein

Representative examples of the tubulointerstitial TG2 protein for Nx, RWI, and RWI+TGI groups are shown in Figures 4D–F, respectively. Cell nuclei, detected by DAPI, are shown in blue. RWI was associated with a higher intense red signal (1.9-fold) in the tubulointerstitial area (Nx, 0.85 ± 0.12; RWI, 1.6 ± 0.05 Alexa/DAPI ratio, *P* < 0.001). TGI significantly reduced the increase in TG2 immunostaining by 91% to values which showed no statistical difference from the Nx control group (RWI+TGI, 1 ± 0.07 Alexa/DAPI ratio, *P* < 0.001). Nx, *n* = 5; RWI, *n* = 5; RWI + TGI, *n* = 6.

Tubulointerstitial TG2 protein gave significant linear correlations with tubulointerstitial fibrosis determined by individual collagens, collagen I (*r*^2^ = 0.38, *P* < 0.01), collagen III (*r*^2^ = 0.53, *P* < 0.01) and collagen IV (*r*^2^ = 0.67, *P* < 0.001). Also, serum creatinine showed a moderate correlation with tubulointerstitial TG2 protein (*r*^2^ = 0.42, *P* < 0.01).

### 3.5 Tubulointerstitial collagens

Representative examples of tubulointerstitial kidney collagen I FITC (green) for Nx, RWI and RWI+TGI groups are shown in Figures 4G–I, respectively; and for collagen III are shown in Figures 4J–L, respectively. Cell nuclei detected by DAPI are shown in blue.

The RWI group was associated with a significant increase (1.8-fold) in collagen I immunofluorescence (Nx, 0.24 ± 0.03; RWI, 0.42 ± 0.06 FITC/DAPI ratio, *P* < 0.05). TGI abolished the increase in collagen I immunostaining (RWI+TGI, 0.19 ± 0.03 FITC/DAPI ratio, *P* < 0.01). Collagen III in the RWI group was significantly increased (4.3-fold) in the interstitial area compared to the Nx group (Nx, 0.12 ± 0.01; RWI, 0.52 ± 0.04 FITC/DAPI ratio, *P* < 001). TGI significantly reduced tubulointerstitial collagen III by 57% compared to the RWI alone (RWI+TGI, 0.34 ± 0.03 FITC/DAPI ratio, *P* < 0.01). Collagen IV in the RWI group was associated with a significant increase (5.5-fold) in the peritubular area compared to the Nx group (Nx, 0.04 ± 0.01; RWI, 0.22 ± 0.02 FITC/DAPI ratio, *P* < 0.001). TGI reduced the increase in collagen IV immunostaining by some 90% (RWI+TGI, 0.06 ± 0.01 FITC/DAPI ratio, *P* < 0.001). Nx, *n* = 5; RWI, *n* = 5; RWI + TGI, *n* = 6.

Individual tubulointerstitial collagens I (*r*^2^ = 0.30), III (*r*^2^ = 0.65) and IV (*r*^2^ = 0.61) showed a positive significant linear correlation with serum creatinine measured at day 28.

### 3.6 HIF-2 alpha

In kidney sections from rats subjected to RWI, notable HIF-2α expression was observed in two out of three rats. Immunoreactivity with strong signal was localized to the intraglomerular mesangial area and the tubular epithelium within both cortical and medullary regions. Due to the limited availability of experimental tissue (*n* = 3), no quantitative comparison between groups was performed.

## 4 Discussion

In the cat, RWI generates renal damage with measurable reduction of renal function, which correlates with histopathological changes of AKI, progressing to CKD (9, 10). Naturally occurring CKD in cats is associated with predominant damage to the tubulointerstitium compared to the glomeruli (14, 28, 29). This pattern of injury has been replicated in the RWI feline model following 70 days of unilateral renal warm ischaemia (9). Similar findings have been shown in feline renal isografts and autografts (30, 31). In the domestic feline with CKD, proteinuria is not a prominent feature in the first stages of renal disease, consistent with a low level of glomerular scarring, which is similar to our RWI model. With more severe damage, a subtle elevation of proteinuria, a marker of CKD progression and prognosis (32), can be seen. In the rat, RWI is the first stimulus demonstrated to inhibit the glomerular TG pathway where, a decrease TG enzyme activity and TG2 protein was associated with decreased intraglomerular collagen. The down regulation of glomerular TG2 by a stimulus which, upregulates the TG pathway in the tubulointerstitium, could indicate the presence of a protective mechanism against mesangial expansion. Glomerular protection may be important due to the low regenerative capacity of the glomerulus compared to the renal tubule (33, 34). A glomerular protection mechanism would be of major interest in diabetic nephropathy in humans, where glomerular scarring poses a significant challenge (5, 35). A nephron cannot function without a glomerulus but, nephron function can be re-established after severe tubular damage due to more effective and faster tubular regeneration (36).

The lack of expansion of the basement membrane (collagen IV) in the mesangial capillary and Bowman's capsule, as well as the low expression of collagen I in the mesangial interstitium is attributed to the proposed protective effect of glomerular hypoxia reducing the baseline collagen content, driven by the reduction in the transglutaminase pathway. This mechanism is proposed to slow future degeneration of the kidney by stimulating the development of functional parenchyma. Cell cycle regulators, such as cyclin dependant kinase 4 (CDK-4) have also been shown to be absent from the glomerulus compared to the tubulointerstitium following RWI in the rat (33). The lack of this cell cycle regulator in the IGMA could therefore also be involved in glomerular protection, but it has yet to be studied in feline CKD.

Hypoxia exerts significantly greater damage on the tubulointerstitial microvasculature than on the glomerular capillary network (37). The hypoxia-inducible factor (HIF) isoforms play distinct roles at different stages of tissue hypoxia, orchestrating adaptive responses including energy conservation, angiogenesis, metabolic reprogramming, and cell survival. These effects are mediated through the activation of a range of target genes that help maintain cellular homeostasis under low oxygen conditions (38). HIF-1α has been primarily associated with early hypoxic responses, particularly promoting angiogenesis and renal inflammation (39–41). This is consistent with its marked transcriptional upregulation observed in a rodent model of acute renal warm ischaemia (RWI) as early as 6 h after reperfusion (42) and also in feline CKD (14). Interestingly, upregulation of HIF-1α in podocytes via SUMO-specific protease 1 has also been shown to exert protective effects on glomerular endothelial cells (37).

In contrast, HIF-2α appears to play a more prominent role during the later stages of hypoxic injury, with anti-inflammatory and anti-fibrotic properties. Although both isoforms are tightly regulated to maintain a physiological balance during hypoxic events, this equilibrium can be disrupted by underlying disease processes. For instance, in diabetic nephropathy, the protective actions of HIF-2α may be attenuated, thereby facilitating disease progression (41). HIF-2α expression has been reported in glomerular cells, cortical peritubular capillaries, and select interstitial cells in rats exposed to systemic hypoxia or renal ischaemia (16). In murine models of RWI and unilateral ureteral obstruction, deletion of HIF-2α leads to increased expression of inflammatory markers, underscoring its critical role in modulating inflammation and promoting tissue recovery (43). Furthermore, appropriate regulation of HIF-2α is essential for preserving the integrity of the glomerular filtration barrier (44). The involvement of HIF-2α in the tubulointerstitial compartment has been more extensively studied than its role in the glomeruli. Notably, several investigations using tetracycline-inducible HIF-2α overexpression models targeting the tubuloepithelial cells in chronic kidney disease and renal warm ischaemia have demonstrated its potential to attenuate renal fibrosis and enhance renal function (43, 45, 46). In our rodent model HIF-2α was colocalized in both mesangial and tubuloepithelial regions. Although quantification was not possible due to limited tissue, this distribution may suggest a relevant involvement of HIF-2alpha as seen in multiple models of HIF-2α and RWI in mice, where its absence led to increased inflammation and impaired tissue recovery (43, 47, 48).

The major isoforms of transglutaminase are factor XIIIa, in blood plasma, and TG2 in kidney tissue from both the rat and domestic feline (7). A causal link between TG2 and tubulointerstitial fibrosis in renal disease has already been established in animal models of TG2 gene ablation and following transglutaminase inhibition (18, 49–52). The present study showed the development of fibrosis in the tubulointerstitium with a significant reduction of collagen deposition by TG inhibition. Reduced TG2 activity decreases the degradation-resistant cross-link dipeptide ε(ɤ-glutamyl)-lysine in collagen proteins, facilitating the breakdown of the deposited collagen in the ECM by natural proteolytic systems (53). This phenomenon would also be expected to reduce the activation of inflammatory and fibrogenic pathways (54, 55). TG2 can upregulate TGF-β transcription and activate TGF-β1 by crosslinking the latent form of TGF-β to the extracellular matrix where it becomes cytokine-activated (50, 54, 56). By inhibiting TG2, a decrease in the intervention of TGF-β in the fibrogenic process could have been induced. The reduction of TGF-β could attenuate TG2 transcription resulting in a decrease in enzyme availability (57). The overexpression of TGF- β has also been shown in a rodent model of RWI (17). This phenomenon may explain why a reduction in extracellular TG2 occurred in our own study following TG inhibition, when protein production is assumed to be a transcriptional event.

TG2 may also contribute to inflammatory events, since TG2 transcriptional inhibition is able to decrease the activation of NF-κβ and COX-2 pathways *in vitro* and *in vivo* (51, 58). NF-κβ activation via TG2 has been shown to be associated with I-κβ kinase dependent and independent pathways (55, 59). The TG inhibitor employed in the current study has an extracellular effect and does not directly influence TG2 transcription. However, other indirect pathways for the reduction of inflammation by TGI have been proposed since the reduction of fibrosis can also be secondary to a reduction of inflammatory stimuli, reducing cellular infiltration, decreasing collagen substrate. In this regard, a role for TGF-β1 in inflammation has been postulated. TGF-β1 gene expression is up-regulated in the rat following RWI in both the outer medulla and tubules; after 14 days, it is just identifiable in regenerating tubules (17). RWI in the rodent model generates an increase in kidney tissue malondialdehyde (peroxidation product) and eicosanoids. During renal inflammation TGF-β1 stimulates fibroblast differentiation through the induction of a pro-oxidant shift in intracellular redox status mediated via reactive oxygen species (60). Therefore, an anti-inflammatory effect of TGI could also be associated with the reduction in the activation of TGF-β1 that lessens the levels of ROS in the interstitial space.

Rat and mouse models of kidney disease, including 5/6th subtotal nephrectomy, unilateral ureteral obstruction (UUO), diabetic nephropathy and drug-induced nephrotoxicity, where hypoxia may play an important role, all generate tubulointerstitial fibrosis (18, 49–52, 61, 62). However, the histopathological features do not match those of feline CKD since a severe form of glomerulosclerosis is also generated. Hypoxia is important to the development of CKD following AKI where, it has been established as a final common pathway for the development of renal fibrosis (11, 12, 63). Regarding postrenal AKI, urethral obstruction has also been recognized as a major cause of AKI in the cat (64). UUO in mice induces vasoconstriction and hypoxia developing lesions compatible to ischaemic injury (65). However, the assessment of UUO, which could be a surrogate model for urethral blockage in the cat, is limited to histopathology rather than functional studies due to the inability to obtain blood serum renal markers and collect urine from the affected kidney.

The rat model of RWI has been widely studied demonstrating an adequate replication of CKD in humans following acute kidney injury (13, 66). Main histopathologic features include tubulointerstitial inflammation, acute tubular necrosis, peritubular capillary lesions, tubular atrophy and fibrosis similar to the renal lesions found in the cat subjected to RWI (9, 10, 67). Tubulointerstitial fibrosis and inflammatory cellular infiltration within the extracellular matrix and tubular atrophy are features found in feline and human CKD, regardless of etiology (9, 29).

AKI generates a sequela which remains in the kidney as a residual passive injury promoting progression of CKD (68, 69). Traditionally, AKI has been divided in intrinsic, prerenal and postrenal injury in both humans and companion animals (70) in which low oxygen tensions are generated. Acute tubular necrosis (ATN) is a key histopathologic feature in both humans (71) and cats (9, 10, 72) of nephrotoxic and intrinsic ischaemic kidney disease, in which close intervention of hypoxia has been postulated. In the cat however, apparently the most common causes of intrinsic AKI were nephrotoxic rather than ischemic stimuli (73). Interestingly, an hypoxemic event can also be secondary to renal toxicity, as some common nephrotoxic etiologies can trigger a decrease in renal perfusion limiting oxygen availability, as seen by NSAID intoxication (74). Prerenal AKI is seen in the veterinary setting in patients with acute hemorrhage, severe dehydration, anaphylactic reactions and hypotension during anesthesia, to mention some causes. Prerenal AKI causes hypovolaemia leading to severe fall in systemic blood pressure generating vasoconstriction in different systems, including the kidney. This auto-regulatory phenomena preserves the basic immediate vital systems to maintain cardiac output and cerebral perfusion, promoting renal hypoxemia (75). Renal hypoxemia appears to play a relevant role in any renal injury contributing to the development of renal fibrosis in both our RWI rodent model and the cat subjected to RWI. The tubulointerstitial fibrosis in our model is consistent with naturally occurring feline CKD further supporting our RWI model as a potential tool to study CKD in this species and perhaps could reduce experimentation in cats (9, 10, 76). Furthermore, the low expression of the renal transglutaminase pathway and fibrosis in the glomeruli contrasted with the high level of tubulointerstitial transglutaminase expression and scarring in rats subjected to RWI is a unique pattern identified in our model, showing similar molecular and histological patterns compared to feline CKD (7).

The activation of the tubulointerstitial TG pathway in our model resulted in the development of tubulointerstitial fibrosis both inhibited by the intrarenal infusion of a TG inhibitor. Sanchez-Lara et al. (7) study showed an effective *in vitro* inhibition of the transglutaminase pathway in feline renal tissue with CKD by radioactive 3H putrescine assay using a selective TG2 neutralizing monoclonal antibody (BB7 antibody). The study showed that effective antibody inhibition is feasible in feline kidney tissue and that TG2 is the major isoform of feline CKD (7). Notably, the BB7 antibody, along with another TG2 inhibitory antibody (DCI), has been humanized and investigated in human *in vitro* studies as well as *in vivo* models of chronic kidney disease in rabbits and cynomolgus monkeys, with the aim of progressing toward clinical trials for targeted TG2 therapy in human kidney fibrosis (77, 78).

This step is highly relevant for Veterinary Science as employing a feline-specific (“catified”) antibody could have a ground-breaking impact in Veterinary Medicine on feline CKD to stop, delay or perhaps even reverse the progression of tubulointerstitial renal fibrosis, the core histopathological renal abnormality in cats with CKD (14). The successful inhibition of the enzyme in our RWI rodent model with reduction of tubulointerstitial fibrosis adds to the compiling evidence showing the TG pathway is a causal link for the development of tubulointerstitial fibrosis and that this model mimics the expression of collagens and TG2 in a similar fashion as on the domestic cat with CKD, opening a door relevant for future therapeutic trials.

A limitation of this study was the absence of electron microscopy, which could have provided additional insights into ultrastructural changes within the glomerular and tubular compartments. Additionally, while histomorphological assessment could have complemented the study, conventional staining techniques such as PAS, H&E, and Masson's trichrome (MTS) may not offer sufficient specificity for early fibrotic changes, have been associated with variable reproducibility, and may be confounded by inflammatory edema—particularly in the case of MTS—leading to potential overestimation of fibrosis (79–82). This consideration is especially relevant given that the renal warm ischaemia (RWI) model is known to induce a pronounced inflammatory response and increased capillary permeability in both the glomerular and tubulointerstitial compartments (25, 83, 84). Considering these factors, and due to the central role of collagen deposition in assessing transglutaminase 2–mediated fibrogenesis, immunostaining targeting specific collagen types was selected. This approach offers improved specificity and enables precise colocalization and quantification of fibrotic alterations between experimental groups (80).

In conclusion, the rat model of RWI exhibits notable molecular and histopathological similarities to CKD in cats, providing a valuable platform to study feline renal disease. The RWI model's characteristic low glomerular transglutaminase pathway and scarring expression support the existence of a protective glomerular mechanism that is worth exploring further. This finding could have profound implications for understanding the minimal glomerular scarring and TG pathway pattern observed in feline CKD and may offer a pathway for studying and treating human glomerulosclerosis. The efficacy of transglutaminase inhibition in mitigating tubulointerstitial fibrosis in the RWI rodent model highlights its potential for translational therapeutic studies in cats with CKD and diabetic nephropathy in humans.

## Data Availability

The original contributions presented in the study are included in the article/supplementary material, further inquiries can be directed to the corresponding author.
